# Sentinel surveillance for bacterial pneumonia and meningitis in children under the age of 5 in a tertiary pediatric hospital in Colombia - 2016

**DOI:** 10.7705/biomedica.5658

**Published:** 2021-10-15

**Authors:** Germán Camacho-Moreno, Carolina Duarte, Diego García, Viviana Calderón, Luz Yanet Maldonado, Liliana Castellar, Jaime Moreno, Jacqueline Palacios, Ángela Gallego, Orlando Castillo, Olga Sanabria, Ivy Talavera, Rubén Montoya

**Affiliations:** 1 HOMI, Fundación Hospital Pediátrico La Misericordia, Universidad Nacional de Colombia, Bogotá, D.C., Colombia Universidad Nacional de Colombia Universidad Nacional de Colombia Bogotá D.C Colombia; 2 Grupo de Microbiología, Instituto Nacional de Salud, Bogotá, D.C., Colombia Instituto Nacional de Salud Bogotá D.C Colombia; 3 Dirección de Promoción y Prevención, Subdirección de Enfermedades Transmisibles, Grupo de Inmunoprevenibles PAI, Ministerio de Salud y Protección Social, Bogotá, D.C., Colombia Subdirección de Enfermedades Transmisibles Grupo de Inmunoprevenibles PAI Ministerio de Salud y Protección Social Bogotá D.C Colombia; 4 Promoción de la Salud y Curso de Vida, Organización Panamericana de la Salud, Bogotá, D.C., Colombia Organización Panamericana de la Salud Bogotá D.C. Colombia; 5 Laboratorio de Salud Pública, Secretaría Distrital de Salud, Bogotá, D.C., Colombia Secretaría Distrital de Salud Bogotá D.C. Colombia

**Keywords:** Sentinel surveillance, pneumonia, meningitis, Streptococcus pneumoniae, Haemophilus influenzae, vigilancia de guardia, neumonía, meningitis, Streptococcus pneumoniae, Haemophilus influenzae

## Abstract

**Introduction:**

Bacterial pneumonia and meningitis are vaccine-preventable diseases. Sentinel surveillance provides relevant information about their behavior.

**Objective:**

To present the data from sentinel surveillance carried out at the *Fundación HOMI, Fundación Hospital Pediátrico La Misericordia* in 2016.

**Materials and methods:**

We conducted a descriptive study from January 1 to December 31, 2016, on the daily surveillance of patients under 5 years of age diagnosed with pneumonia or bacterial meningitis according to PAHO's definitions. We identified the microorganisms using the automated VITEK^TM^ 2 system. Bacterial isolates were sent to the Microbiology Group at the Colombian *Instituto Nacional de Salud* for confirmation, serotyping, phenotypic, and genotypic characterization. Antimicrobial susceptibility profiles were established.

**Results:**

From 1,343 suspected cases of bacterial pneumonia, 654 (48.7%) were probable, 84% had complete Hib vaccination schedules, and 87% had complete pneumococcal vaccination schedules for age. Blood culture was taken in 619 (94.6%) and 41 (6.6%) were positive while *S. pneumoniae* was isolated in 17 (41%) of them. The most frequent serotype was 19A in five cases (29.4%), and four 19A serotypes were associated with the reference isolate ST320. The incidence rate of probable bacterial pneumonia was 7.3 cases/100 hospitalized patients, and lethality was 2.1%. As for bacterial meningitis, 22 suspected cases were reported, 12 (54%) were probable, four (33%) were confirmed: two by *Escherichia coli* and two by group C *N. meningitidis*. The incidence of probable bacterial meningitis was 0.14 cases/100 hospitalized patients.

**Conclusion:**

*Streptococcus pneumoniae serotypes* 19A and 3 were the most frequent cause of pneumonia. Spn19A is related to the multi-resistant clone ST320. Strengthening and continuing this strategy will allow understanding the impact of vaccination.

## Resumen

**Introducción.:**

La neumonía y la meningitis bacterianas son enfermedades inmunoprevenibles; la vigilancia centinela aporta información relevante acerca de su comportamiento.

**Objetivo.:**

Presentar los resultados de la vigilancia centinela de neumonía y meningitis llevada a cabo en la HOMI, Fundación Hospital Pediátrico La Misericordia.

**Materiales y métodos.:**

Se hizo un estudio descriptivo entre el 1 de enero y el 31 diciembre del 2016, de la vigilancia diaria de pacientes menores de 5 años con diagnóstico de neumonía o meningitis bacteriana, según las definiciones de la Organización Panamericana de la Salud (OPS). Los microorganismos fueron identificados usando el sistema automatizado VITEK ^TM^2. Los aislamientos se enviaron al grupo de microbiología del Instituto Nacional de Salud para confirmación, serotipificación, y caracterización genotípica y fenotípica. Asimismo, se establecieron los perfiles de sensibilidad antimicrobiana.

**Resultados.:**

De 1.343 casos sospechosos de neumonía bacteriana, 654 (48,7 %) fueron probables, el 84 % tenía el esquema de vacunación completo para la edad contra *Haemophilus influenzae* de tipo b, y el 87 %, contra neumococo. En 619 (94,6 %) pacientes se hizo hemocultivo y 41 (6,6 %) fueron positivos. S*. pneumoniae* se aisló en 17 (41 %) casos. El serotipo más frecuente fue el 19A, en cinco pacientes (29,4 %), en tanto que cuatro aislamientos de spn19A fueron relacionados con el clon ST320. La tasa de incidencia de neumonía bacteriana probable fue de 7,3 casos/100 pacientes hospitalizados. La letalidad fue de 2,1 %. Hubo 22 casos sospechosos de meningitis bacteriana, 12 (54 %) probables, y cuatro (33 %) confirmados: dos por *Escherichia coli* y dos por *Neisseria meningitidis* del grupo C. La incidencia de meningitis bacteriana probable fue de 0,14/100 pacientes hospitalizados.

**Conclusión.:**

Los serotipos 19A y 3 de *S. pneumoniae* fueron la causa más frecuente de neumonía. El Spn19A se relacionó con el clon ST320 mulitirresistente. El fortalecimiento continuo de la vigilancia centinela permitirá entender el impacto de la vacunación.

Pneumonia is one of the most common causes of hospital admissions and death in children under five years of age. In developed countries, viruses are considered the leading cause of pneumonia while the bacterial etiology is proportionally higher in developing countries. The most common causative agents are *Streptococcus pneumoniae*, *Haemophilus influenzae* type b, *Moraxella catarrhalis*, and *Staphylococcus aureus*^1^^-^^4^.

Bacterial meningitis, although not as common as pneumonia, is a severe disease with high mortality rates and risk of sequelae. The most frequent agents are *S. pneumoniae, H. influenzae* type b, and *Neisseria meningitis*^2^^,^^5^. In Colombia, bacterial meningitis is a notifiable disease, and pneumonia is targeted for collective surveillance ^6^^,^^7^. In this context, sentinel surveillance of bacterial pneumonia and meningitis contributes to generating standardized and timely data to characterize the epidemiological behavior of these diseases in children under five years of age. Moreover, it provides data to measure vaccination status and the impact of vaccines on morbidity and mortality ^8^^,^^9^.

To initiate the epidemiological surveillance of bacterial pneumonia and meningitis, measure disease burden and the impact of pneumococcal conjugate vaccine (PCV) introduction, and to determine the circulation of serotypes and changes in the susceptibility to antibiotics, the World Health Organization implemented the Global Invasive Bacterial Vaccine- Preventable Diseases (IB-VPD) Laboratory Network. The objective of this work is to present the data from sentinel surveillance carried out at the *HOMI, Fundación Hospital Pediátrico La Misericordia*.

## Materials and methods

### Case definitions

From January 1 to December 31, 2016, daily surveillance was performed on patients under five years of age admitted to the institution with a diagnosis of pneumonia (International Classification of Diseases Code ICD -10 J10 - J18) or bacterial meningitis (International Classification of Diseases Code ICD -10 A321, A390, G000, G001, G002, G003, G008, G009, G01X, G028, G038, G039). For epidemiological surveillance of acute bacterial pneumonia (ABP), we considered the following definitions established by the Pan American Health Organization (PAHO):

- Suspected case of pneumonia: Every patient under the age of 5 hospitalized with a diagnosis of community-acquired pneumonia. A hospitalized patient is any patient that requires inpatient treatment.
- Probable case of bacterial pneumonia: Any suspected case with a chest x-ray showing a radiological pattern compatible with bacterial pneumonia.
- Confirmed case of bacterial pneumonia: Any probable bacterial pneumonia case in which *H. influenzae, S. pneumoniae*, or other bacteria were identified or cultured in blood or pleural fluid.
- Discarded case of bacterial pneumonia: Any suspected case with no chest X-ray showing a radiological pattern compatible with bacterial pneumonia.
- Inadequately investigated case of pneumonia: Any suspected case without a chest x-ray.

We reviewed patients' records every day. Chest x-rays were performed to screen suspected cases, two peripheral blood cultures were taken from probable cases and, in the presence of pleural effusion, a culture of the pleural fluid was taken. Pediatric radiologists interpreted chest X-rays according to the World Health Organization (WHO) manual of diagnostic imaging ^10^.

We considered all children under five years of age hospitalized with a medical diagnosis of meningitis as a suspected case of meningitis; these patients underwent lumbar puncture and blood cultures. The case was considered as probable if the cerebrospinal fluid (CSF) had any of the following characteristics: Turbidity, increased leukocytes (> 100/mm3), or leukocytes between 10-100/mm3, and elevated protein (> 100 mg/dL), or low glucose levels (< 40 mg/dl). All suspected cases with a bacterium recognized for causing meningitis in blood or CSF were considered as confirmed cases. Discarded cases included those suspected with CSF cytochemical values not compatible with meningitis and negative cultures. Finally, any suspected case without a CSF sample was considered inadequately investigated.

We collected the clinical data for all patients included in the surveillance, as well as the information from their vaccination cards, and when they did not have them, we systematically searched for the records in the Expanded Immunization Program (PAI) website (web https://paiweb.gov.co.) and Bogota's web page (www.saludcapital.gov.co/Pai/publico/busqueda.aspx).

### Identification of microorganisms

The sentinel institution identified the microorganisms isolated in cultures using the automated VITEK^™^ 2 system (bioMerieux, Marcy I'Etoile, France) while bacterial isolates were sent to the Microbiology Group at the *Instituto Nacional de Salud* by Bogota's public health laboratory for confirmation, and phenotypic and genotypic characterization. The pneumococcal isolates were serotyping by Quellung reaction, serogroup of *N. meningitidis*, and the serotype of *H. influenzae* was determined by the slide agglutination assay using commercial antisera (DIFCO, Becton Dickinson). We determined the antimicrobial susceptibility profiles using the disk diffusion test (Kirby- Bauer) and microdilution in broth to determine resistance to penicillin (PEN), ampicillin (AMP) ceftriaxone (CRO), trimethoprim-sulfamethoxazole (SXT), chloramphenicol (CHL), tetracycline (TET), erythromycin (ERY), and rifampicin (RIF). Results were interpreted based on the criteria of the Clinical & Laboratory Standards Institute (CLSI) 2016 standards ^11^. Multidrug resistance (MDR) was defined as resistance to three or more antibiotic families.

We conducted additional studies to those suggested in the protocol for a better characterization of the isolated agents. For the genotypic characterization, we used pulsed-field gel electrophoresis (PFGE) according to the protocol by Vela, *et al*. ^12^ and the R6 strain as a control. We used the Spain^9V^-ST156, Colombia^23F^-ST338, and Netherlands^3^-ST180 clones, as well as the representative isolates of ST199, ST276, ST320, ST460, and ST473 sequence types, as electrophoretic reference standards. We analyzed PFGE patterns with GelCompar II version 4.0 using the unweighted pair group method with arithmetic mean (UPGMA) and the Dice coefficient with optimization and tolerance of 1.5% to generate a genetic similarity dendrogram. PFGE patterns with similarity over 75% were grouped as a clonal group and designated with capital letters.

### Sentinel center

The *HOMI*, *Fundación Hospital Pediátrico La Misericordia* is a private tertiary referral hospital located in Bogotá, which treats children with bacterial pneumonia and bacterial meningitis. Between 2011 and 2015, 206 cases of invasive pneumococcal disease (IPD) in children under five years of age were reported in Bogotá, 48 (23%) of them by *Fundación HOMI*^13^. In 2016, the Ministry of Health and Social Protection chose the hospital to be the sentinel surveillance center for bacterial pneumonia and bacterial meningitis, as it meets the inclusion criteria to be part of the network and has the capacity to respond at the care, epidemiological, and laboratory levels. To date, it is the only of its kind in the country ^14^.

### Selection bias

We identified the risk of selection bias in the study since it was conducted in a tertiary care hospital where patients with more severe diseases are admitted. We also acknowledged the probability of bias in the analysis of the chest X-rays. There was also the possibility of bias risk due to the loss of microbiological isolates during the process for which we developed a protocol for sending the samples and verifying their viability upon arrival at their destination.

### Statistical analysis

This was a descriptive study. We collected the data in an Excel database, performed frequency analyses of the variables, and estimated the average length of stay in the intensive care unit (ICU) and the incidence of pneumonia and meningitis per 100 hospital admissions.

### Ethical considerations

This was a risk-free study since the patients did not undergo any intervention, procedure, or test besides those indicated for the diseases. It was approved by the Ethics and Research Committee at the *HOMI, Fundación Hospital Pediátrico La Misericordia*.

## Results

During the study period, 1,343 suspected cases of bacterial pneumonia were captured; 654 (48.7%) met the probable case criteria, 380 (58%) were children under 2 years of age, 358 (55%) were male, and 296 (45%), female. Table 1 shows the general characteristics of patients with pneumonia. The most frequent symptoms and signs in probable cases were fever (94%), cough (92%), respiratory distress (82%), subcostal retractions (51%), cyanosis (28%), and vomiting (25%). Thirty-seven cases (5.6%) received antibiotic treatment the week before hospitalization.

**Table 1:** Sociodemographic characteristics of probable pneumonia cases (N=654)

Variable	n (%)
Sex	
Male	358 (55)
Female	296 (45)
Age (months)	
<2	13 (2)
2 - 11	181 (27.6)
12 - 23	184 (28.1)
24 - 59	276 (42.2)
Number of probable cases with blood culture	619 (94.6)
Number of confirmed cases	41 (6.6)
Mortality	14 (2.1)

Among the 654 probable cases, there were 13 children under the age of 2 months; of the 641 patients older than 2 months, 539 cases (84%) had complete Hib vaccination schedules for age (pentavalent vaccine), 61 (9.5%) had incomplete schedules, and 41 (6.4%) had no vaccination records. Finally, 559 cases (87%) had complete pneumococcal vaccination schedules for age, 41 (6.4%) had incomplete schedules, and 41 (6.4%) had no vaccination records. All patients vaccinated against pneumococcus received PCV10.

Table 2 shows the serotype and the doses of pneumococcal and *H. influenzae* type b vaccine administered to the confirmed cases. Most of the patients with pneumococcal isolation had received at least 2 doses of the vaccine, while three with serotypes 14, 3, and 19A had received 3 doses of PCV10. It was not possible to obtain vaccination data for the only case with *H. influenzae* type b isolate. Among the probable cases (n=654), the most frequent radiological findings were consolidation (54.5%), other alveolar opacities (40.2%), pleural effusion (3.2%), and interstitial opacities (1.9%). Of these cases, 185 (28.2%) were admitted to the intensive care unit (ICU) with an average stay of five days.

**Table 2:** List of confirmed cases of bacterial pneumonia due to *Streptococcus pneumoniae* and *Haemophilus influenzae* and vaccination status

Confirmed case	Age in months	# of Hib vaccine doses	# of PCV10 vaccine doses	Serotyping
*Streptococcus* *pneumoniae*	29	3	3	19A
	40	3	3	19A
	35	3	3	19A
	7	2	2	19A
	31	ND	ND	19A
	22	3	3	3
	11	3	2	3
	10	3	2	3
	18	3	3	3
	30	3	3	14
	41	3	3	14
	38	3	3	14
	41	3	2	14
	11	3	2	9N
	8	3	2	9N
	21	3	3	6A
	40	3	3	15A
*Haemophilus* *influenzae*	3	1	1	NT
	3	1	1	NT
	42	3	2	NT
	59	ND	ND	NT
	55	ND	ND	NT
	4	ND	ND	B
	36	3	3	A
	2	1	1	ND
*Neisseria* *meningitidis*	4	1	1	Group C
			

*ND: No data; NT: Non-typeable

**Figure 1:**
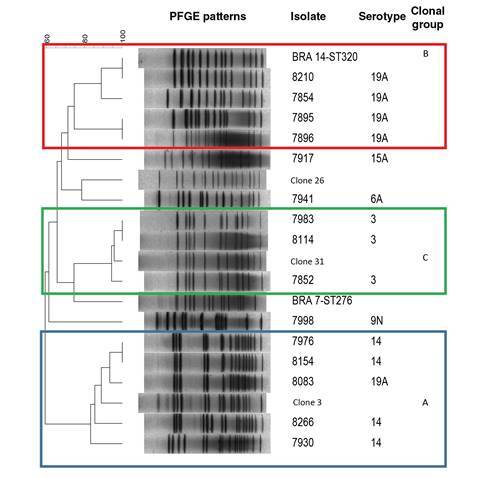
Dendrogram of the genetic relationship between *Streptococcus pneumoniae* isolates

Blood culture was done in 619 (94.6%) probable cases, 41 (6.6%) of which were positive; *S. pneumoniae* was isolated in 17 (41%) of them (table 3). All *S. pneumoniae* isolates were serotyped; the most frequent serotype was 19A with 5 (29.4%) cases, followed by serotypes 3 and 14 with 4 (23.5%) cases, respectively. Isolates with type 19A capsules were resistant to PEN, CRO, STX, ERY, and TET; serotype 14 was resistant to PEN, CRO, and STX while isolates with serotypes 3, 6A, 9N, and 15A were susceptible to all antibiotics tested (table 4). The 16 isolates analyzed by PFGE were grouped into three electrophoretic groups (A, B and C) (table 4, figure 1). Group A, consisting of four serotype 14 and one 19A isolates was related to the Spain9V-ST156 clone; group B grouped four serotype 19A isolates and the reference isolate ST320, and group C consisted of the capsular type 3 isolates and the Netherlands3-ST180 clone. The remaining isolates were not electrophoretically related.

**Table 3:** Microorganisms most frequently isolated with bacterial pneumonia

Microorganism	n	%
*Streptococcus pneumoniae*	17	41.5
*Haemophilus influenzae*	8	19.5
*Staphylococcus aureus*	4	9.8
*Escherichia coli*	3	7.3
*Moraxella catharralis*	1	2.4
*Streptococcus pyogenes*	1	2.4
*Klebsiella pneumoniae*	1	2.4
*Neisseria meningitidis*	1	2.4
*Enterobacter aerogenes*	1	2.4
*Pantoea agglomerans*	1	2.4
*Proteus mirabilis.*	1	2.4
*Serratia marcescens*	1	2.4
*Sphingomonas paucimobilis*	1	2.4
Total confirmed cases	41	100.0

**Table 4:** Phenotypic and genotypic characteristics of *Streptococcus pneumoniae* isolates recovered from children with pneumonia

Isolate	Minimum inhibitory concentration (μg/ml)									
	PEN	CRO	CHL	SXT	ERY	VAN	TET	**Serotype**	PFGE pattern	**Clone**
GMR-Spn7854	4	1	2	>4	>1	0.50	>8	19A	B	ST320
GMR-Spn7895	4	2	4	4	>1	0.50	4.	19A	B	ST320
GMR-Spn7896	4	2	4	4	>1	0.50	>8	19A	B	ST320
GMR-Spn8083	4	2	4	4	>1	0.50	>8	19A	A	ST156
GMR-Spn8210	8	2	4	>4	>1	0.50	>8	19A	B	ST320
GMR-Spn7852	0.03	0.016	2	0.125	0.06	0.50	0.25	3	C	ST180
GMR-Spn7855	0.03	0.03	2	0.125	0.06	0.5	0.25	3	C	ST180
GMR-Spn7983	0.03	0.016	2	0.125	0.06	0.5	0.25	3	C	ST180
GMR-Spn8114	0.03	0.03	2.	0.125	0.03	0.5	0.25	3	C	ST180
GMR-Spn7930	2	1	2	>4	0.06	0.5	0.5	14	A	ST156
GMR-Spn7976	4	2	4	4.	0.06	0.5	0.5	14	A	ST156
GMR-Spn8154	4.	2	4	>4	0.06	0.5	0.5	14	A	ST156
GMR-Spn8266	4	2	4	4	0.06	0.5	1	14	A	ST156
GMR-Spn7997	0.03	0.03	2	0.25	0.06	0.5	0.25	9N	NR	
GMR-Spn7998	0.03	0.03	2	0.25	0.06	0.5	0.25	9N	NR	
GMR-Spn7941	0.25	0.06	4	0.125	0.06	0.5	0.25	6A	NR	
GMR-Spn7917	0.06	0.06	4.	0.25	0.06	0.5	0.25	15A	NR	
**Antibiotic**			**CLSI 2016: Minimum inhibitory concentration (μg/ml)**							
			**Susceptible**			**Intermediate**			**Resistant**	
Penicillin (PEN) No meningitis			≤ 2.0			4.0			≥ 8	
Ceftriaxone (CRO) No meningitis			≤ 1.0			2.0			≥ 4	
Chloramphenicol (CHL)			≤4.0						≥8.0	
Trimethoprim sulfa (SXT)			≤ 0.5			1.0 - 2.0			≥ 4	
Erythromycin (ERY)			≤0.25			0.5			≥1.0	
Vancomycin (VAN)			≤1.0							
Tetracycline (TET)			0.016 - 1			2			≥ 4	

NR: Not related

*Haemophilus influenzae* was isolated in eight (19.5%) of the 41 patients with positive cultures; of them, five (62.5%) were non-typeable, one (12.5%) was serotype b, and one (12.5%) was serotype a while no serotype was obtained in one case (12.5%). All isolates were sensitive to AMP, CRO, SXT, RIF, CHL, and TET. *N. meningitis* serogroup C was found in a patient diagnosed with pneumonia and meningitis. There were three cases of empyema, *S. aureus* was documented in two of them, and the culture was negative in the third patient.

During the study period, 8,557 children under 5 years of age were hospitalized in the institution. The incidence rate of clinical pneumonia (suspected cases) was 15.2 cases per 100 hospitalized patients while the incidence rate of probable bacterial pneumonia was 7.3 cases per 100 hospitalized patients. Finally, 14 (2.1%) of the 654 probable cases died; *S. pneumoniae* serotype 3 was isolated in two of these patients and the remaining 12 patients had negative cultures.

As for bacterial meningitis, 22 suspected cases were reported, of which 17 (77%) were under 2 years of age. Of the suspected cases, 12 (54%) were probable and 7 (75%) were patients under 2 years of age. Of the 12 probable cases, four (33%) were confirmed: two by *E. coli* and two by group C *N. meningitidis*. None of the cases had received meningococcal vaccination. The incidence of probable bacterial meningitis was 0.14 cases per 100 hospitalized patients.

## Discussion

We present here the data from the first year of sentinel surveillance of meningitis and pneumonia in children under 5 years of age treated at the *HOMI, Fundación Hospital Pediátrico La Misericordia*,, essential to understand the epidemiological behavior of these two vaccine-preventable diseases and determine the prevalence of circulating pneumococcal serotypes in the period of study.

As of 2016, the Global Invasive Bacterial Vaccine-Preventable Diseases Laboratory Network has included 58 countries, among them Colombia, and 123 sentinel hospitals, such as the *HOMI, Fundación Hospital Pediátrico La Misericordia*. The objectives of this network are to collect information to describe the epidemiology and burden of vaccine-preventable bacterial invasive diseases, implement a surveillance system to measure the impact of vaccine introduction (Hib or PCV), and detect and characterize circulating serotypes.

In 2006, Colombia introduced the vaccination against pneumococcus with PCV7 ^15^ for children under 2 years of age at high risk and with specific diseases. In 2008, Bogotá extended the strategy by including children weighing 2000 g or less at birth; in October that year, the vaccine was made universal in the city. Then, in 2009, coverage was extended to all children born on or after January 1 residing in the departments with the highest proportion of deaths from acute respiratory infection ^16^.

Later, in the period 2010-2011, due to the withdrawal of the heptavalent vaccine, the PCV13 vaccine was acquired to continue with the immunization of the target population, as well as to complete and finish schemes initiated with the heptavalent vaccine. In 2011, the PCV10 vaccine was universalized in the country and began to be administered in January 2012 to the population born on or after November 1, 2011, using a two-dose schedule and boosters at 2, 4, and 12 months of age. This vaccine is currently administered with 89% coverage including a booster administered in 2016.

Pneumonia is the leading cause of death from infectious diseases in children under 5 years worldwide and accounts for 15% of all deaths, mostly in developing countries ^17^. According to the literature, *S. pneumoniae, H. influenzae, S. aureus*, and the influenza virus are the most frequent causes of pneumonia ^1^^-^^4^^,^^18^, which coincides with the findings of this study.

Furthermore, we found that 48.7% of the suspected cases met the diagnostic criteria of probable case. This percentage is higher than that reported in a previous study conducted by the *Saludcoop* hospital network in Bogotá, with 35.1% probably due to the inclusion of patients aged 0-36 months only, in whom the prevalence of viral cases is higher ^19^.

Regarding clinical findings, fever was the most common symptom similar to the reports of other studies ^20^; 82% of the patients showed signs of respiratory distress, which, together with hypoxia, have been considered sensitive clinical signs for the diagnosis of pneumonia ^21^.

The WHO pneumonia surveillance protocol is based on chest X-ray results ^22^ whose sensitivity in some studies has been 85% and 93% for the diagnosis of community-acquired pneumonia ^23^^,^^24^, while other studies show a lower sensitivity (34.3%) ^25^. To reduce interobserver variability for detecting alveolar consolidation and pleural effusion, for our study, we held a meeting with the radiology group to standardize these definitions ^24^^,^^26^^-^^28^. Consequently, the most frequent radiological finding was consolidation, consistent with the findings of other studies ^19^^,^^29^.

The percentage of positive blood cultures (6.6%) was similar to that found by Lakhani, *et al*. (6.1%) and Jain, *et al*. (8%) ^23^^,^^30^, and higher than that in Benavides, *et al*. previous study in Bogotá (1.5%)^19^, as well as that found by Shah, *et al*. (2.1%) ^31^, Obaro, *et al*. (3.1%) ^32^ and Davis, *et al*. (1.1%) ^33^. This is probably related to the implementation of appropriate incubation methods, such as the CO_2_ chamber and the use of automated methods for the identification of microorganisms. The number of patients admitted to the ICU (28.2%) was similar to that reported by Tiewsoh, *et al*. (20.8%) ^34^ and by Jain, *et al*. (21%) ^30^ while mortality from pneumonia was lower (2.1%) than that reported by Tiewsoh, *et al*. (10.5%) ^34^ and higher than that reported by Jain *et al*. (<1%) ^30^^,^^34^.

The clinical pneumonia incidence rate was 15.2 cases per 100 hospitalized patients while probable bacterial pneumonia was 7.3 cases per 100 hospitalized patients. Between 2009 and 2015, 31% of hospitalizations of children under 5 years in Colombia were associated with acute respiratory infections (ARI) (J00-J22) including some codes (J00-J09 and J19-J22) that are not subject to sentinel surveillance ^35^.

A study conducted in Bogotá in healthcare delivery centers of the Saludcoop network before the systematic administration of the conjugate vaccine found an incidence of clinical pneumonia of 6,276 cases/100,000 patients under 36 months and confirmed pneumonia through imaging in 2,120 cases/100,000 patients under 36 months ^19^. Another study conducted in Brazil reported an incidence of clinical and radiologically confirmed pneumonia of 9,598/100,000 cases and 3,428/100,000 cases, respectively ^36^. Data from these two studies are not comparable with those of the present study as different denominators were used.

Mortality from ARI in Colombia decreased between 2007 and 2016 from 24 to 13.9 cases per 100,000 children under 5. In Bogotá, the trend has been similar with a decrease in ARI mortality from 6.1 to 4.3 per 100,000 children under 5 ^35^^,^^37^.

In our study, 53% of the *S. pneumoniae* isolates were resistant to one or more antibiotics and 29.5% were multidrug-resistant. Over the past two decades, *S. pneumoniae* has become increasingly resistant to several antibiotics including cephalosporins, macrolides, and fluoroquinolones, and estimates are that 20-30% of pneumococcal disease cases worldwide are multidrug-resistant ^38^._

Our results show that serotypes 3, 14, and 19A, which are genetically related to international *S. pneumoniae* clones, are a leading cause of pneumonia in children under 5 ^39^. Serotype 14 isolates, mostly associated with the Spain9VST156 clone in the pre-PCV era, have been considered the main cause of the invasive pneumococcal disease ^39^. In a study conducted in Bogotá before the introduction of the conjugate vaccine, the most frequent serotypes were 14, 19B, and 6B ^19^. The prevalence of serotype 14 in children under 5 has declined in recent years, from 29.1% in 2006 to 6.1% in 2016 ^40^ possibly as a result of the use of conjugate vaccines as reported in other countries ^41^.

As in Parra, *et al*.'s ^39^ study, we identified a 19A capsule variant, probably explained by an adaptive response to the selective pressure exerted by the conjugate vaccine. Isolates of multidrug-resistant *S. pneumoniae* serotype 19A associated with the spread of ST320, a variant of the international clone Taiwan19F14-ST236, have been reported worldwide and constitute one of the most frequent causes of pneumococcal disease ^42^.

A meta-analysis reaffirmed the predominant contribution of 19A to invasive pneumococcal disease in children from different regions in the world after the introduction of PCV7 as it was identified as the most common serotype in cases from the Americas, Europe, and Western Pacific regions ^43^. A multicenter study conducted in 10 hospitals in Bogotá reported an increase of serotype 19A among children under 5 from 4.7% in 2008-2011 to 36.8% in 2014-2017 ^44^. Furthermore, a time-series study using data on *S. pneumoniae* serotypes causing IPD in children under 5 from 1994 to 2016 published by the National Health Institute ^40^^,^^45^ found that the annual proportion of serotypes 6A, 19A, and 3 remained constant until 2012. Subsequently, a more than two-fold increase was observed. The time-series analysis exposed a relationship between the introduction of PCV10 and the increased proportion of 19 A and 3 serotypes with coefficients of 20.92 (p=0.00, ARIMA (2,0,1)) and 6.32 (p=0.00, ARIMA (2,1,1)), respectively ^40^^,^^45^. These data differ from a recent systematic review including data up to 2015 ^46^. Significant reductions of 19A have been observed in the United States after 5 years of PCV13 use ^43^, findings also reported in the United Kingdom ^47^, Canada ^48^, Denmark ^49^, Israel ^50^, and France ^51^.

Serotype 3 isolates were associated with the Netherlands3-ST180 clone, which in turn is associated with 95% of invasive Colombian *S. pneumoniae* isolates carrying this capsular type ^52^. Phenotypically, this clone is susceptible to antimicrobials but penicillin and erythromycin-resistant isolates have also been reported ^53^.

As for bacterial meningitis, the proportion of probable cases compared to suspected ones was 54% higher than the reports by Ramachandran, *et al*. in India (12.5%) ^54^. Most cases were children under 2, similar to the findings from other studies ^55^. During the study period, 12 cases of probable meningitis were treated at the sentinel hospital. There were no cases of meningitis due to *S. pneumoniae or H. influenzae* type b while two cases of meningitis due to *N. meningitidis* were reported. In 2016, 19 cases of *S. pneumoniae*, 23 of H. influenzae type b, and 38 of *N. meningitidis* meningitis in children under 5 were reported to the national surveillance system ^56^.

One of the strengths of our study was the coordinated participation of all actors responsible for epidemiological surveillance: the Ministry of Health and Social Protection, the *Instituto Nacional de Salud*, the Pan American Health Organization (PAHO), and the *Secretaría Distrital de Salud de Bogotá*. Other strengths are the prospective collection of data and the characteristics of the sentinel hospital, which serves a significant number of the city's patients with IPD and has the infrastructure and epidemiological support to ensure good data quality. Study requirements were met, and patient recruitment and analysis complied with PAHO standards.

Regarding limitations, the study was conducted in a tertiary care hospital in Bogotá and the data may not be extrapolated to other populations. Another limitation was the difficulty in obtaining vaccination data for all patients because the information system (which is the source of the data) was not up to date. There may also be biases derived from radiological interpretation. Finally, this study does not allow determining the impact of the vaccines as this requires sustained surveillance data for a 3 to 5 year period.

*Streptococcus pneumoniae* is the most common cause of pneumonia while the most frequent serotypes were those not included in PCV10 (Spn19A, Spn3). Serotypes 19A and 14 are multi-resistant. The second most common bacterium was non-typeable *H. influenza* and one case of *H. influenza* type b was reported. There were two cases of meningitis due to group C *N. meningitidis*. Epidemiological sentinel surveillance is a strategy that provides insight into the epidemiological behavior of vaccine-preventable diseases. Strengthening and continuing this strategy will allow a better understanding of vaccination impact.
